# Vertebral Augmentation Compared to Conservative Treatment of Vertebra Plana and High-Degree Osteoporotic Vertebral Fractures: A Review of 110 Fractures in 100 Patients

**DOI:** 10.7759/cureus.22006

**Published:** 2022-02-08

**Authors:** David M Joyce, Michelle Granville, Aldo Berti, Robert E Jacobson

**Affiliations:** 1 Pain Management, Larkin Community Hospital, Miami, USA; 2 Neurosurgery, University of Miami Hospital, Miami, USA; 3 Neurosurgery, Miami Neurosurgical Center, Miami, USA

**Keywords:** high degree vertebral fractures, vertebra plana, osteoporosis, spinejack, vertebral compression fracture, vertebroplasty

## Abstract

This is a retrospective study that evaluated surgical versus non-surgical treatment of 100 patients followed for up to six years diagnosed with severe osteoporotic vertebral compression fractures (VCF). Fractures were classified by percent collapse of vertebral body height as "high-degree fractures" (HDF) (>50%) or vertebra plana (VP) (>70%). A total of 310 patients with VCF were reviewed, identifying 110 severe fractures in 100 patients. The HDF group was composed of 47 patients with a total of 50 fractures. The VP group was composed of 53 patients with a total of 60 fractures. Surgical intervention was performed in 59 patients, comprised entirely of percutaneous vertebral cement augmentation procedures, including vertebroplasty, balloon kyphoplasty, or cement with expandable titanium implants. The remaining 41 patients only underwent conservative treatment that is the basis of the comparison study. All procedures were performed as an outpatient under local anesthesia with minimal sedation and there were no procedural complications. The initial or pre-procedural visual analog scale (VAS) score averaged 8.4 in all patients, with surgical patients having the most marked drop in VAS, averaging four points. This efficacy was achieved to a greater degree in surgically treated VP fractures compared to HDF. Non-surgical patients persisted with the most pain in both short- and long-term follow-up. This large series, with follow-up up to six years, demonstrated that the more severe fractures respond well to different percutaneous cement augmentation procedures with reduction of pain without increased complications in a comparison to conservatively treated patients.

## Introduction

Most of the osteoporotic vertebral compression fractures are related to falls or load injuries on an underlying osteoporotic spine in elderly patients. Many less severe compression fractures, between 10% and 30% collapse, heal spontaneously or with conservative bracing with a short interval of pain. However, severe osteoporotic compression fractures with greater than 50% collapse in vertebral height are important to recognize and separate from minor vertebral compression fractures since they have an increased risk of continued progressive collapse, spinal angulation, and evolution to vertebra plana (VP) if left untreated [1]. We further sub-divide severe fractures into two groups: high-degree fractures (HDF), defined as fractures with 50-70% collapse; and VP, defined as fractures with greater than 70% collapse. This range of severe vertebral compression fractures are more commonly seen at the thoracolumbar junction, which serves as the fulcrum of thoracolumbar spinal motion, creating biomechanical instability and increasing the risk for progressive collapse even with bracing. It is generally theorized that the greater the percentage of vertebral collapse, the greater the resultant spinal kyphotic angulation, which shifts the center of gravity anteriorly making the patient vulnerable to fracture progression, presenting as worsening collapse, increased kyphosis, higher risk for adjacent level fractures, and predisposing patients to multiple "cascading" fractures [2]. 

Patients with continually painful osteoporotic vertebral fractures and patients with or without pain that present with progressively worsening fractures should be recommended for surgery, despite conservative treatment or bracing. Surgery can involve either open reduction, corpectomy with multilevel pedicle screw fixation, or more recently short segment fixation combined with vertebroplasty, which has been recommended to try and correct the kyphosis, or different types of percutaneous vertebral augmentation [3,4]. Open surgery can be problematic in these elderly patients since the screws will often not hold well in softer osteoporotic bone and elderly patients typically have significant medical comorbidities, increasing their risk of complications from blood loss and time under general anesthesia. Over the last 20 years, there have been multiple smaller series demonstrating that osteoporotic vertebral fractures can be treated using various minimally invasive percutaneous techniques such as vertebroplasty, balloon kyphoplasty, and most recently expandable implants. All of these can be performed under local anesthesia, often in an outpatient setting [5,6]. Despite large experience with percutaneous treatment of fractures, VP fractures have often been listed as a contraindication for vertebral augmentation assuming it was technically difficult or impossible to cannulate the severe compression, and that percutaneous augmentation procedures would not provide sufficient expansion, height restoration, and reduction of kyphosis compared to open surgery [1,2,7-9].

This is a retrospective review of 100 patients found to have a total of 110 severe osteoporotic vertebral fractures with greater than 50% vertebral collapse. These severe fractures were identified from a larger group of 310 patients with osteoporotic compression fractures seen over a six-year time period in a private neurosurgical practice. Fractures were radiographically assessed with either magnetic resonance imaging (MRI) and/or computerized tomography (CT) scans. If there was a question regarding the acuteness of the fracture, a nuclear medicine bone scan was ordered, and bone mineral density (BMD) was evaluated with dual-energy X-ray absorptiometry if prior studies were not available within 24 months preceding the patient's initial complaint. Of the 310 identified patients with osteoporotic vertebral compression fractures, 53 patients (17%) were diagnosed with VP and an additional 47 patients (15.2%) had HDF with greater than 50% collapse. All patients were treated either surgically with different percutaneous vertebral augmentation procedures or were treated conservatively with bracing and medication management. Treatment decision was made by the patient and family after explanation of all options, including a clear explanation of radiographic findings and risks. This large-series review examined the specific type of procedure performed, outcomes of surgical intervention, and also a comparison in outcome between surgical treatment and non-surgical treatment. Additionally, the review examined the time interval between an inciting injury and diagnosis, and the time to surgical intervention. 

Demographically, both the surgical treatment group and the conservative treatment group were similar. The severity of osteoporosis, and the associated factors including age, chronic use of corticosteroids, and a history of previously diagnosed osteoporotic limb and osteoporotic vertebral compression fractures were evaluated. Pre- and post-procedure visual analog scale (VAS) scores in both conservative and operative patients were compared. The review of similar patients with HDF enabled comparison of treatment outcomes between various minimal vertebral augmentation procedures and conservative treatment.

## Materials and methods

A retrospective chart review of all patients with vertebral compression fractures of two neurosurgeons in a single neurosurgical office, spanning January 2016-December 2021, was done.

Three hundred and ten patients were selected using an ICD-10 code M80.08 (age-related osteoporosis with current pathological fracture, vertebra(e)). Using radiological measurements based on MRI or CT, the degree of collapse was assessed in the sagittal plane, comparing measurements of vertebral body height at the maximum point of collapse in millimeters (mm) to the height of the posterior vertebral border in mm. One hundred of 310 patients were identified as having severe vertebral compression fractures with greater than 50% collapse. A total of 110 severe vertebral compression fractures were identified in this group of 100 patients. 

These 100 patients with 110 fractures were further divided into two groups for comparison: an HDF group, presenting with fractures quantified as having greater than 50% collapse without VP (N=47), and a second VP group presenting with fractures greater than 70% collapse (N=53).

The two cohorts were then evaluated to identify differences or similarities between the groups. Electronic medical records, paper charts, and digital imaging were thoroughly reviewed to look at the following variables: 1) age and gender, 2) type of surgery, 3) VAS score, 4) vertebral fracture level, 5) time lapse between inciting injury and surgery, 6) time lapse between diagnosis and treatment, 7) previous fracture history, 8) previous spinal surgery, 9) bone mineral density, 10) pharmacological treatment for osteoporosis, 11) medical comorbidities.

## Results

We examined pertinent patient demographics including age, gender, osteoporotic fracture history, vertebral compression fracture history, chronic prednisone use, and anticoagulation or antiplatelet therapy. These variables were compared between the surgical and non-surgical groups, which were very similar in all categories. The average number of days from inciting injury to diagnosis, diagnosis to surgery, and total time from injury to surgery were relatively the same between both the HDF and VP patients who underwent vertebral augmentation. Twenty of 26 HDF patients and 23 of 32 VP patients were able to recall the date of their initial injury. Time from injury to diagnosis averaged 48 vs 43 days; diagnosis to surgery averaged 31 vs 35 days; and total time from injury to surgery was 79 days in both groups (Table 1). 

**Table 1:** Matched data sets n = total number of patients per group; Age = average age; Female = number of female patients; Male = number of male patients; Fx Hx = osteoporotic fracture history, all fractures; VCF Hx = vertebral compression fracture history; Steroid = chronic steroid use (only daily prednisone included); Anticoag = antiplatelet and/or anticoagulation therapy (ASA 81 mg excluded)

Surgery (n=58)		No Surgery (n=42)	
Age	81	Age	82.1
Female	43 (75%)	Female	35 (83%)
Male	14 (25%)	Male	7 (17%)
Fx Hx	15 (26%)	Fx Hx	16 (38%)
VCF Hx	9 (16%)	VCF Hx	8 (19%)
Steroid	6 (11%)	Steroid	4 (10%)
Anticoag	13 (23%)	Anticoag	12 (29%)

**Figure 1:**
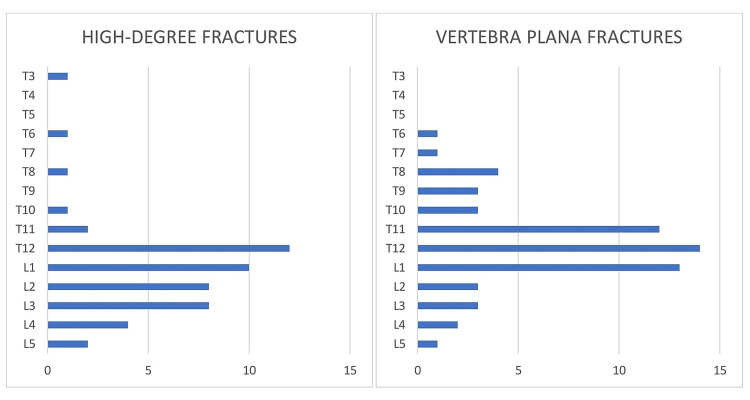
Fracture distribution graph comparison T = thoracic; L = lumbar

Using radiological measurements of the patient's MRI or CT, a total of 110 severe vertebral compression fractures with greater than 50% collapse were identified in 100 patients. Forty-seven patients (47%) had HDF (50-70% collapse), not classified as VP and 53 patients (53%) were found to have VP fractures (>70% collapse) at the time of diagnosis of vertebral compression fracture. Of the 47 HDF patients identified, 26 underwent vertebral augmentation (55.3%) and 21 patients did not undergo surgery (44.7%). Of the 53 patients with VP, 32 underwent vertebral augmentation (60.4%) and 21 patients did not undergo surgery (39.6%). The distribution was graphed, and in both groups, the highest percentage of fractures were in the thoracolumbar transition zone between T11 and L1, although there was a significantly higher number of T11 and T12 fractures, finding 26/53 (49%) in VP vs 14/47 (29.8%) in the HDF group. In contrast, lumbar fractures were identified in 32/47 (68%) of the HDF group compared to 22/53 (41.5%) in the VP group. If we included L1 in the thoracolumbar junction (T11 to L1) then there were 24/47 (51%) fractures in the HDF group and 39/53 (73.5%) in the VP group (Figure 1). 

Type of surgery: In the HDF group, 26 patients underwent a total of 28 vertebral augmentation procedures (13 vertebroplasties, 4 balloon kyphoplasty, 11 SJ^R^), as shown in Table 1. Two patients had revision surgeries on single-level fractures because of persistent pain or continued vertebral collapse. One patient underwent balloon kyphoplasty and had revision vertebroplasty. A second patient underwent balloon kyphoplasty and had revision SJ^R^. In the VP group, 32 patients underwent a total of 34 vertebral augmentation procedures (18 vertebroplasties, 5 balloon kyphoplasty, 11 SJ^R^). Two patients each had two-level VP fractures and each underwent vertebroplasty and kyphoplasty on the same operative day, respectively. 

VAS: Scores were averaged separately within the HDF and VP groups, according to patients who underwent vertebral augmentation and those who did not have surgery, respectively. In the HDF group, there was a 5.6-point reduction in patients who underwent surgery versus a 2.1-point reduction in patients who did not have surgery. In the VP group, there was a 4.7-point reduction in patients who underwent surgery versus a 0.3-point reduction in patients who did not have surgery. Patients who underwent balloon kyphoplasty for HDF had a 6.3-point VAS reduction and patients who underwent SJ^R^ implants for VP fractures had a 6.3-point VAS reduction (Table 2 and Table 3). 

**Table 2:** VAS comparison table for HDF patients VAS = visual analog score, averaged by patients who reported both pre-operative and post-operative VAS; VP = vertebroplasty; KP = kyphoplasty; SP = SpineJack implant; n = number of patients; Sx = surgical group; No Sx = non-surgical group

Patients With High-Degree Fractures					
	Sx (n=26)				No Sx (n=21)
	VP (n=13)	KP (n=4)	SJ (n=11)	All Surgeries	Total
Pre-VAS	7.5	7.8	8.9	8.1	7.9
Post-VAS	2.4	1.5	3.4	2.4	5.7
Delta	5.1	6.3	5.5	5.6	2.1

**Table 3:** VAS comparison table for VP patients VAS = visual analog score, averaged by patients who reported both pre-operative and post-operative VAS; VP = vertebroplasty; KP = kyphoplasty; SJ = SpineJack implant; n = number of patients; Sx = surgical group; No Sx = non-surgical group

Patients With Vertebra Plana Fractures					
	Sx (n=32)				No Sx (n=21)
	VP (n=18)	KP (n=5)	SJ (n=11)	All Surgeries	
Pre-VAS	7.2	8	8	7.7	6
Post-VAS	2.7	4.5	1.7	3	5.7
Delta	4.5	3.5	6.3	4.7	0.3

VAS was also averaged separately between all patients who underwent vertebral augmentation (n=57) versus all patients who did not undergo surgical intervention for similar types of severe vertebral compression fractures (n=42). Surgical patients showed an average VAS reduction of 3.4 points, while non-surgical patients showed an average VAS reduction of 1.3 points (Table 4). 

**Table 4:** VAS comparison between all surgeries versus non-surgical patients Pre-VAS (surgery) = VAS at initial office visit; Pre-VAS (no surgery) = VAS at initial office visit; Post-VAS (surgery) = VAS at post-operative visit; Post-VAS (no surgery) = VAS at final office visit; VAS Delta = reduction in VAS; VAS = visual analog scale score

Surgery (n=58)		No Surgery (n=42)	
Pre-VAS	8.1	Pre-VAS	6.9
Post-VAS (First Post-op Visit)	2.6	Post-VAS (No sx)	5.6
VAS Delta	5.5	VAS Delta	1.3

## Discussion

From a database and retrospective chart review of 310 patients who were evaluated over six years, we subdivided vertebral fractures into under 50% collapse and over 50% collapse as measuring by comparison of the posterior vertebral height of the fractured vertebra to the maximum sagittal collapse. A group of 100 patients with greater than 50% collapse were identified. These 100 patients had 110 acute and subacute high-degree osteoporotic fractures which is the subject of this report. This group of patients was then further subdivided into fractures with collapse between 50% and 70% which we designated as HDF and those over 70% defined in the literature as VP fractures [2-4]. These cases were selected only from a referral neurosurgical practice seeing patients after their initial evaluation by family medicine, internal medicine, orthopedics, or pain management because of persistent symptoms, degree of fracture, multiple fractures, or complicating co-morbidities. As a result, these 310 cases did not necessarily reflect a true number or percentages of fractures with severe collapse during the time period since this did not include any emergency cases or cases of minor compression fractures with minimal pain that returned to primary care following evaluation and improved with conservative treatment and were not referred to our office. Treatment options offered to the patients were conservative bracing and physical therapy or having one of several different percutaneous vertebral augmentation procedures. Of the 100 patients who met the greater than 50% fracture criteria, 58 were treated with percutaneous vertebral augmentation and 42 conservatively which is the basis of the comparison of the treatment outcomes in this group of patients. As shown previously in Table 1, the demographic data were very similar for age, initial VAS scores, time to diagnosis, and comorbidities, providing the opportunity to make a clear comparison of outcomes. Patients who chose conservative treatment did so primarily because either the patient and/or family did not want any recommended surgical procedure or because of medical comorbidities that prevented medical clearance. Advanced age or use of anticoagulants did not exclude patients from surgery unless the patient or family declined, or clearance was denied. The improvement in VAS scores was more marked in the patients undergoing interventional cement injection with the greatest treatment efficacy seen with insertion of expandable implants. This finding was previously reported on a study of use of the SJ^R^ implants in treating VP fractures [8,9].

Radiographic studies using plain X-rays, CT, and MRI demonstrated a continuous range of progressive collapse in these fractures in some patients from mild (10% or 20% compression) to more severe compression and angulation, eventually evolving to VP. There were no distinctive differences in those "presenting" with HDF or VP fractures versus the overall patient population, except that the severe fractures were concentrated in the thoracolumbar junction between T11 and L1 and the patients were slightly older than the average. The VP fractures included more cases at T11 and the HDF group included more lumbar fractures. In many cases, the patients presented at the initial visit with either HDF or VP fractures, commonly with a six-week or greater gap between initial injury and definitive radiologic diagnosis. 

**Figure 2:**
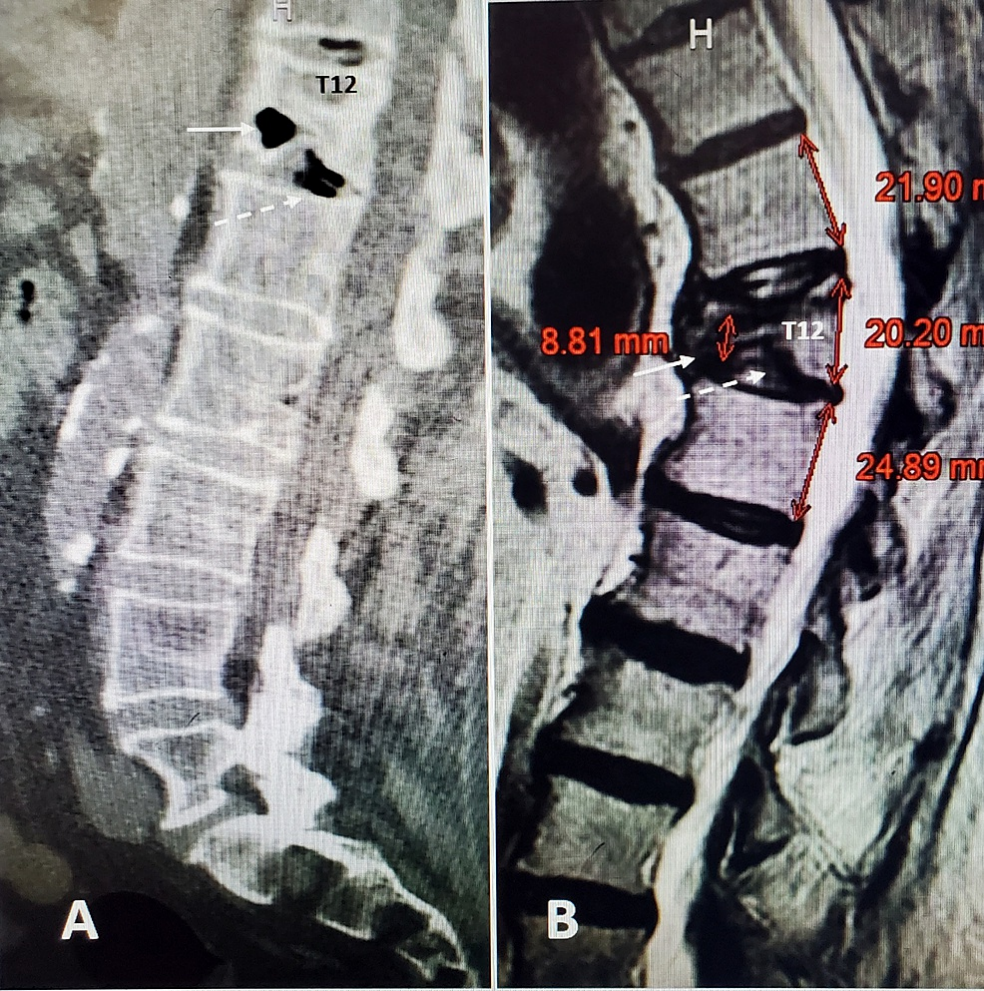
Initial CT scan with vacuum changes at T12 fracture and follow-up MRI showing progressive collapse to vertebra plana and kyphosis A: CT scan three weeks after fall in a 78-year-old female. There is no deformity but severe vacuum changes (solid white arrows) in both the anterior vertebra and T12-L1 disc space. B: MRI scan six weeks alter medical clearance but before surgery showing progressive collapse anteriorly and now there is 15 degrees of kyphotic deformity. The posterior vertebral wall of T12 is 20 mm compared to 22 and 24 of the adjacent vertebral bodies but the anterior part of T12 has collapsed to 8 mm resulting in a 67% collapse.

**Figure 3:**
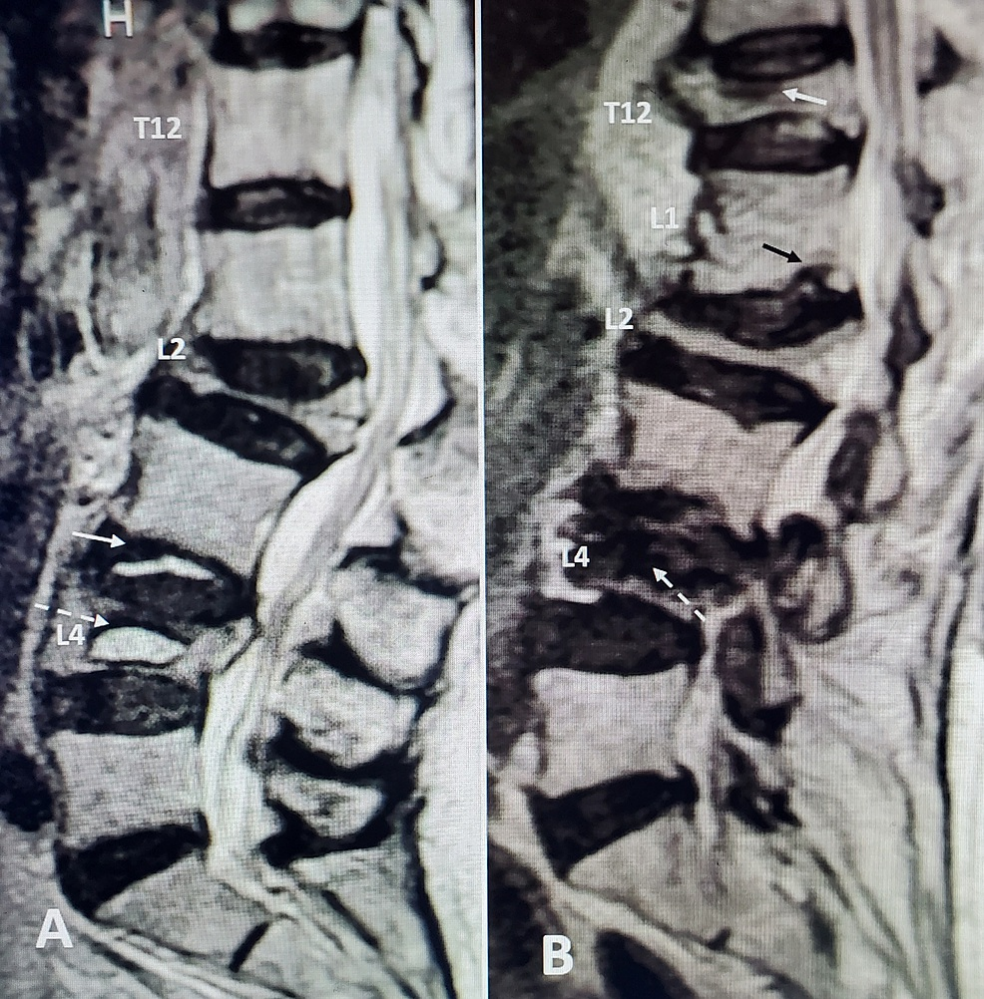
Multiple high-degree and vertebra plana fractures seen over a 24-month period of time A: Initial MRI when patient only had lumbar pain due to L4 high-degree fracture with vertebral clefts (solid white arrow) indicative of both acute fracture and micro-instability. Although the L2 vertebra plana was present three years previously, there was no pain at that level and a bone scan did not show uptake at L2 but only at L4. The L4 fracture was treated with balloon kyphoplasty. B: The same patient re-evaluated for thoracic pain 18 months later. There is a new T12 70% "pancake" fracture with almost complete collapse under superior endplate of T12 (solid white arrow). There is a small inferior endplate fracture posteriorly at L1 (solid black arrow). The old L2 fracture is unchanged. The L4 fracture with black signal bone cement is partially re-expanded (dashed white arrow).

These elderly patients often have significant medical comorbidities, such as cardiac and pulmonary disease, diabetes on multiple medications, and long-term use of anticoagulants for atrial fibrillation or venous embolic disease, so prolonged time getting medical clearance can lead to further delay in providing interventional treatment for the fracture. When we examined the time to surgery there was no obvious difference between the HDF and VP groups, so it is not clear, although delay can allow fracture progression or even development of additional fractures that are found at the time of intra-operative fluoroscopy. Of note, approximately 26% of patient charts reviewed did not identify a specific date for the onset of original symptoms for the fracture. As a result, we feel at minimum, follow-up radiographs, if not a new CT or MRI, should be performed before any surgical procedure to assess the status of the original fracture that may have progressed and to detect the development of any new fractures. Both progression and new fractures can develop without another clear incident or obvious change in symptoms such as spinal pain. Our informal "rule" is if there is a significant time interval of at least four to six weeks between initial films and neurosurgical evaluation or scheduled procedure then it is better to obtain new images (Figure 2 and Figure 3).

The surgical treatment of symptomatic osteoporotic fractures has evolved from open surgical correction with multi-level pedicle screw fixation, requiring general anesthesia and multiple-day hospital stay, to more minimally invasive percutaneous vertebroplasty, balloon kyphoplasty, or internal expansion devices such as the SJR, all combined with use of polymethyl methacrylate (PMMA) bone cement [4-9]. This study demonstrates that VP fractures can be safely and routinely treated percutaneously. Many elderly patients have various comorbidities and present with compression fractures with a high percentage of collapse. Percutaneous treatment for these severe fractures is effective and these procedures can be performed with local anesthesia, possibly with minimal sedation as needed in an ambulatory or outpatient setting. In our review of 100 cases, 13 patients were on various oral chronic anticoagulants that were stopped for the procedure without any intraoperative or post-operative bleeding. All surgical patients underwent the percutaneous vertebral augmentation under local anesthesia or with monitored anesthesia care as an outpatient except one with chronic thrombocytopenia (platelet count 110,000) who was undergoing three-level kyphoplasty from T11 to L1 and was kept for 23-hour observation. There were no infections, post-procedure bleeding, or neurologic complaints in any case following surgery although three patients had mild-to-moderate radiating rib pain lasting several weeks with lower thoracic procedures.

When considering surgical treatment for severe osteoporotic vertebral fractures, there are two distinct factors to consider, one clinical and one radiological. First, persistent or worsening spinal pain despite bracing and analgesic medication which is clinical; and second, radiologic progressive fracture collapse with or without development of kyphotic spinal deformity. Progressive collapse can occur with even minimal or no pain, so serial follow-up films are essential in these osteoporotic patients. Neurologic compression is rare in osteoporotic fractures compared to acute traumatic fractures although radicular complaints are reported with lower lumbar fractures and thoracic radicular pain reported with thoracic fractures [4-8]. When evaluating CT or MRI with deformity associated with the fracture there are several measurements and angles which should be evaluated. These include the percentage or degree of collapse measured by comparing the maximal collapse in the sagittal plane to the posterior vertebral wall height which may show slight collapse compared to adjacent vertebral bodies as seen in Figure 1. The actual angle of the vertebral collapse can be measured, which is different from the overall spinal angulation [10,11].

Many of these elderly patients also have separate lumbar degenerative spondylosis, stenosis, or spondylolisthesis, so they may also have separate or pre-existing mid and lower lumbar pain in addition to the acute fracture pain. This pre-existing lumbar degenerative disease can be aggravated by change in both spinal position and limited mobility that results from stiffness and change in posture after the fracture. In our experience it is important to identify any concurrent lumbar degenerative findings, especially previous lumbar pain since this may complicate post-procedure pain scoring and may necessitate planning for subsequent intervention to target radiculopathy or pre-existing neurogenic claudication due to distinctively different spine pathology. Although some pain relief after vertebral augmentation is noted within the initial 24-48 hours, our experience has been to see gradual improvement over seven to 10 days. Initial pain relief is most likely due to stabilization of the fracture when a vertebral cleft is present, or the effect of the heat generated by the exothermic reaction of over 100^o ^C when the PMMA cement cures affecting the basilar-vertebral nerve complex which innervates both endplates. As mentioned, pre-existing and concurrent fractures can be aggravated and be a cause of "residual pain" after successful vertebral augmentation, so this must always be considered when a patient has not had pain relief or had initial relief after vertebral augmentation and now the pain has recurred. Even with resolution of pain, follow-up CT or MRI is necessary because further collapse and deformity can still occur and has been shown to be related to overall morbidity and survival rate with osteoporotic fractures [2,3].

**Figure 4:**
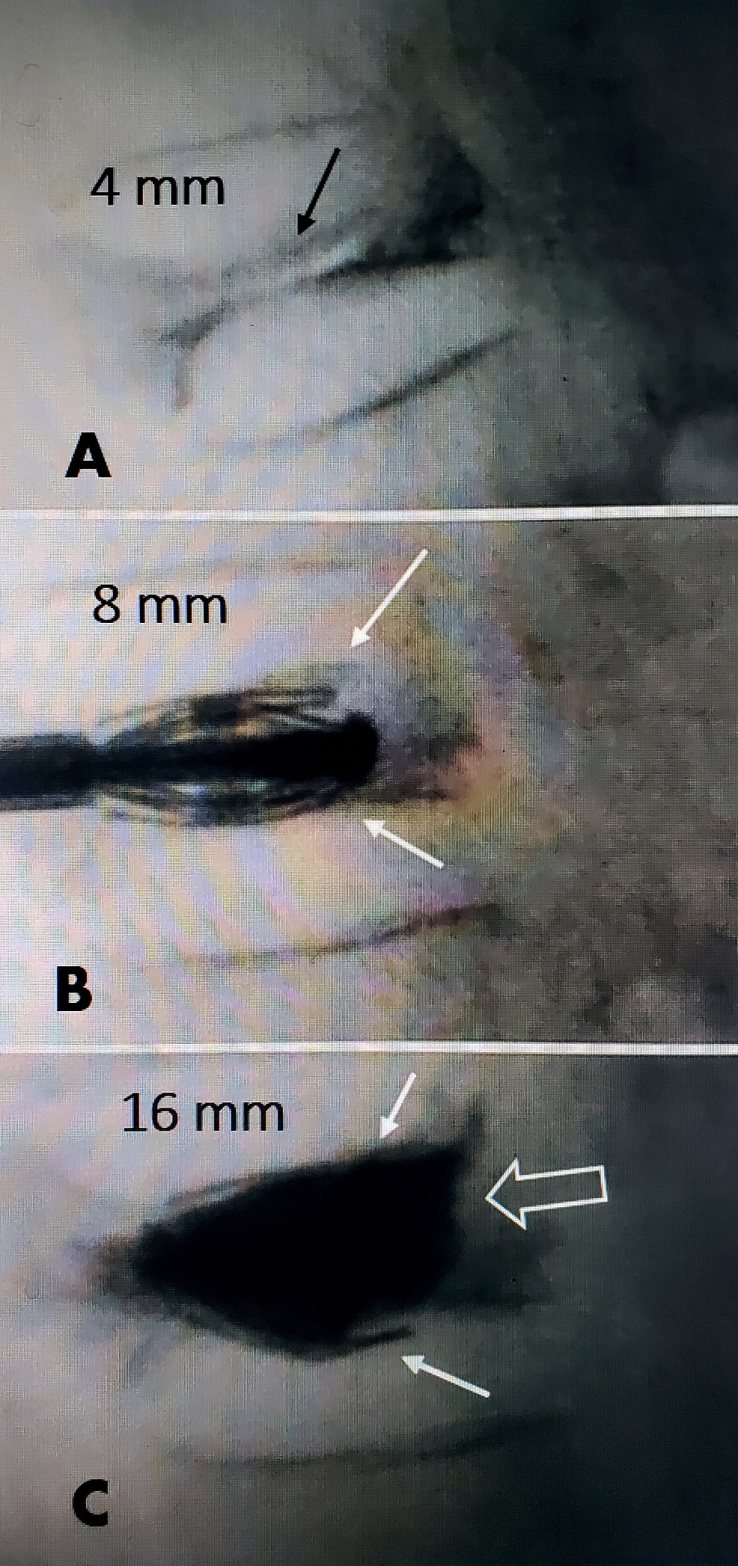
Intra-operative expansion of a vertebral plana A: Severe central biconcave vertebra plana of L1 with only 4 mm residual height. A central vertebral cleft can be seen (black arrow). B: Initial partial expansion of a 4.2 mm expandable implant to 8 mm. The wings of the implant are seen superiorly and inferiorly (solid white arrows). C: Final expansion of the implant to 16 mm height at mid vertebrae (white arrows), the PMMA cement fills the entire body with good expansion anteriorly (open white arrow).

It was originally recommended that anatomic correction of thoracic kyphosis required corpectomy combined with pedicle screw fixation involving two to three segments above and below the fracture as performed with traumatic, non-osteoporotic fractures. Poor bone quality made this construct difficult, so more recently manufactured fenestrated screws allow cement to spread, better anchoring the screws in osteoporotic bone above and below the fracture site to correct the deformity and prevent further collapse. However, these cases were complicated by perioperative morbidity due to advanced age, blood loss, general anesthesia, infection, and risk of loosening of the screw construct because of the underlying poor bone density. Cannulated screws allowing injection of bone cement to provide better screw purchase help the latter problem [6]. With the introduction of percutaneous vertebral access, either by vertebral augmentation with vertebroplasty or combined with balloon expansion, the procedure became much simpler and could restore a degree of kyphotic collapse and vertebral height. Over time, however, the improvement in angle and degree of collapse often regressed, so the added use of titanium expandable implants has helped to reduce this problem. As percutaneous treatments for osteoporotic fractures are significantly less complicated and can be performed under local anesthesia with minimal sedation, the overall risk is greatly reduced as demonstrated by no perioperative morbidity in this series. Generally, less severe degrees of collapse were originally treated percutaneously, and VP was actually listed as a contraindication for percutaneous procedures [6-9]. Anatomically, however, with VP, the vertebral collapse is anterior and more central, so even in the classic severe "flattened" VP, the posterior vertebral wall is held up by the ventral edge of the pedicles so the lateral and posterior sides of the vertebrae rarely collapse allowing access into the collapsed vertebral body with various sized cannulas as long as they are aligned with the plane of the collapse, allowing either cement, a balloon, or an internal expandable strut implant to be placed [12-14]. Severe osteoporotic compression fractures are dynamic, especially when there is the presence of a vertebral cleft. It has been documented that these clefts change in shape and size and fill with flexion and extension, so targeted filling of the cleft with cement is critical in obtaining vertebral height expansion and preventing further collapse [15,16] (Figure 4).

There are specific surgical and technical considerations and modifications when treating HDF and VP fractures with any of the percutaneous methods. It should be routine to compare intra-operative fluoroscopy to the patient's most recent CT or MRI to make sure there are no new fractures or any fracture progression. As previously mentioned, these HDF and VP fractures can change dimension with extension and flexion, so patient positioning can affect the fracture image. Good-resolution MRI and CT scans are essential to determine if the fracture has changed and to evaluate which side, on axial or coronal views, has the greatest collapse. In the surgical or radiographic suite, proper patient positioning with clear imaging of the fracture site is important. Since these patients can be severely osteoporotic, often there is very poor live intra-operative imaging, so exact vertebral count is important to make sure the correct vertebral level is treated. Many of these patients also have separate lumbar degenerate scoliosis or spondylolisthesis, so this can distort the angles for imaging as well as the approach to the pedicle.

Applying both local anesthesia to the skin and along the cannula insertion site and using a long-acting anesthetic like bupivacaine in the periosteum of the bone reduces or eliminates local surgical pain. This is combined with monitored sedation anesthesia. In vertebroplasty cases, we plan for bilateral pedicle access, although often vertebral fill is adequate via a unilateral approach, especially with the use of a curved nitinol curette to create a cavity across the midline. SJR implants are always inserted bilaterally. The anesthesiologist needs to understand that the most painful or uncomfortable part of the procedure is during the impacting of the cannula into the pedicle to help them facilitate appropriate timing of sedation. This is even more critical if using larger-bore cannulas for insertion of expandable implants. The normal vertebral cannulas used for vertebroplasty or balloon kyphoplasty vary from 8 to 11 gauge, which converts to 3.0 mm to 4.2 mm, while the cannulas used in expandable implants range from 4.2 mm to 5.8 mm. The approach can be directly transpedicular or more lateral coming at the junction of the transverse process and pedicle base allowing insertion of even larger cannulas as shown in Figure 4. Our approach is to insert first into the most severe area of collapse documented by MRI or reconstructed CT scans. This ensures that this area is effectively treated, especially in the rare situation when the patient can not tolerate a longer procedure time or becomes medically unstable. Another important radiologic variable is the identification of the presence of a vertebral cleft on CT or MRI. Clefts have been associated with osteonecrosis and intra-vertebral micromotion [14,15]. Intra-operative fluoroscopy in these cases has demonstrated change in size and vertebral height expansion with extension or even distraction at the time of surgery indicating that these severely collapsed vertebrae can be re-expanded [9,15,16]. In cases with clefts the possibility of change in vertebral compression with extension can create greater expansion with a balloon or titanium implant, which in turn increases the vertebral height and lessens the anterior collapse and angulation [3,6,7]. These percutaneous procedures stabilize the fracture, provide partial height and deformity correction, and provide a significant degree of pain relief. 

The comparison of pre- and post-VAS scores comparing non-surgical to surgical treatment of these fractures clearly shows better pain reduction in patients undergoing these procedures. The patients with VP started with slightly higher VAS scores and had more marked reduction (a larger delta) between pre- and post-VAS scores and all vertebral augmentation procedures had better outcomes than the non-surgical patents with demographics and co-morbidities being very similar between all the groups.

## Conclusions

Osteoporotic vertebral fractures form a spectrum between the initial often mild endplate fracture progressing to severe collapse and VP with or without spinal angulation and deformity. The patient can develop more severe fractures and vertebral collapse if there is underlying severe osteoporosis and there has been delay from onset to radiologic diagnosis to ultimate treatment. Factors that affect the degree of collapse are location, especially at the thoracolumbar junction, if there are vertebral clefts, and kyphotic angulation of the vertebral column. These severe fractures with greater than 50% collapse in height are more common at the thoracolumbar junction. Medical comorbidities except for chronic steroid use had no effect unless it caused a delay in preoperative evaluation and clearance. Delay in initial diagnosis and treatment of these fractures may be another significant factor in unrecognized progression to HDF and VP. This review clearly demonstrates that these fractures can be safely treated with a variety of more minimal vertebral augmentation procedures. The patients routinely had significant relief of pain with marked reduction in VAS score as well as some deformity correction with minimal perioperative risk. In this group of 100 patients, those undergoing interventional vertebral augmentation did better with both short-term and long-term pain relief than non-surgically-treated patients.
